# A-6G and A-20C Polymorphisms in the Angiotensinogen Promoter and Hypertension Risk in Chinese: A Meta-Analysis

**DOI:** 10.1371/journal.pone.0029489

**Published:** 2011-12-28

**Authors:** Wei Gu, Jielin Liu, Qiuli Niu, Hao Wang, Yuqing Lou, Kuo Liu, Lijuan Wang, Zuoguang Wang, Jingmei Zhang, Shaojun Wen

**Affiliations:** 1 Department of Hypertension Research, Beijing Anzhen Hospital, Beijing Institute of Heart Lung and Blood Vessel Diseases and Capital Medical University, Beijing, People's Republic of China; 2 Emergency Center of Heart, Lung and Blood Vessel Diseases, Beijing Anzhen Hospital, Capital Medical University, Beijing, People's Republic of China; 3 Member of International Society of Hypertension (ISH); University Institute of Social and Preventive Medicine, Switzerland

## Abstract

**Background:**

Numerous studies in Chinese populations have evaluated the association between the A-6G and A-20C polymorphisms in the promoter region of angiotensinogen gene and hypertension. However, the results remain conflicting. We carried out a meta-analysis for these associations.

**Methods and Results:**

Case–control studies in Chinese and English publications were identified by searching the MEDLINE, EMBASE, CNKI, Wanfang, CBM, and VIP databases. The random-effects model was applied for dichotomous outcomes to combine the results of the individual studies. We finally selected 24 studies containing 5932 hypertensive patients and 5231 normotensive controls. Overall, we found significant association between the A-6G polymorphism and the decreased risk of hypertension in the dominant genetic model (AA+AG vs. GG: P = 0.001, OR = 0.71, 95%CI 0.57–0.87, P_heterogeneity_ = 0.96). The A-20C polymorphism was significantly associated with the increased risk for hypertension in the allele comparison (C vs. A: P = 0.03, OR = 1.14, 95%CI 1.02–1.27, P_heterogeneity_ = 0.92) and recessive genetic model (CC vs. CA+AA: P = 0.005, OR = 1.71, 95%CI 1.18–2.48, P_heterogeneity_ = 0.99). In the subgroup analysis by ethnicity, significant association was also found among Han Chinese for both A-6G and A-20C polymorphisms. A borderline significantly decreased risk of hypertension between A-6G and Chinese Mongolian was seen in the allele comparison (A vs. G: P = 0.05, OR = 0.79, 95%CI 0.62–1.00, P_heterogeneity_ = 0.84).

**Conclusion:**

Our meta-analysis indicated significant association between angiotensinogen promoter polymorphisms and hypertension in the Chinese populations, especially in Han Chinese.

## Introduction

Essential hypertension (EH), the major section (over 95%) of hypertension, represents a serious health problem all over the world. In China, hypertension affects more than 18.8% of the adult population, and a total of 170 million people suffer from this disease [1]. Hypertension has a multi-factorial origin arising from an interaction between susceptibility genes and environmental factors [2]. It is noteworthy that about 20% to 60% of the inter-individual variation of blood pressure (BP) is determined by heritable factors [3]. As a consequence, many potential genes involved in blood pressure regulation have been screened and recognized as candidates for hypertension.

The renin-angiotensin-aldosterone system (RAAS) plays a crucial role in the maintenance of BP [4]. Among the system, the angiotensinogen (AGT) is a liver protein that interplays with renin to produce angiotensin I, the prohormone of angiotensin II, which is the major effector molecule of RAAS. The human AGT gene is located on chromosome 1 (1q42–q43) and contains 5 exons [5]. Many variants in the AGT gene can modify the plasma AGT concentration that is directly linked with arterial blood pressure [6]. At present, we paid particular attention to the rs5051 (A-6G) and rs5050 (A-20C) single nucleotide polymorphisms (SNPs) in the promoter region, which both were reported to influence AGT transcriptional activity and then plasma AGT [6]. Haplotype analysis has revealed that the two SNPs, A-6G and A-20C, were in strong linkage disequilibrium [7].

In a 2008 meta-analysis by Pereira et al. [8], the relationship between hypertension and the A-6G and A-20C polymorphisms has been evaluated in European Caucasian subjects. However, no meta-analysis of these specific genetic assocations has been conducted in Chinese populations so far. The genetic background difference between the two ethnic groups may lead to different conclusions. In addition, the published results of Chinese case-control studies for both polymorphisms remained unsettled. Some studies implied that the A-6G polymorphism [9]–[12] as well as A-20C polymorphism [13] in the AGT gene were associated with the increased or even reduced risk of EH in Chinese, whereas most studies [14]–[24] still provided equivocal or largely negative evidence for this relationship. Taken together, we decided to perform a carefully designed meta-analysis from all eligible case-control studies, in order to clarify the role of the A-6G and A-20C polymorphisms in hypertension among the Chinese populations.

## Materials and Methods

### Identification and eligibility of relevant studies

To search for all the studies that examined the association of the A-6G and A-20C polymorphisms with hypertension in Chinese, we conducted a computerized literature search of the PubMed, EMBASE, CNKI (China Nation Knowledge Infrastructure Platform), Wanfang, CBM (China Biological Medicine Database) and VIP databases (up to May 2011), using the following keywords and subject terms: ‘AGT or Angiotensinogen’, ‘polymorphism’, ‘hypertension’ and ‘Chinese or China or Taiwanese or Taiwan’. We only included studies published in Chinese or in English. References of retrieved articles were also screened. When a report overlapped with another publication, to prevent data duplication, only the more detailed one was kept. If an article reported results on different ethnic sub-populations, each sub-population was treated as separate study in our meta-analysis. We used the following inclusion criteria for a study to be included in the meta-analysis: (a) studies investigating the association of the A-6G or A-20C polymorphisms with hypertension in Chinese individuals, (b) use of an unrelated case–control design (family-based study design with linkage considerations was excluded), (c) available genotype frequency, (d) the genotype distribution of the control population must be in Hardy–Weinberg equilibrium (HWE) and (e) hypertension defined as systolic blood pressure (SBP)≥140 mmHg and/or diastolic blood pressure (DBP)≥90 mmHg and/or treatment with anti-hypertensive medication. If the genotype frequency was not reported, we contacted the original authors by e-mail in order to obtain the missing data.

### Data extraction

Two authors (W. Gu and J. Liu) independently reviewed and extracted the data needed. Disagreements were resolved through discussion among the authors to achieve a consensus. The following information was abstracted from each study: first author, year of publication, racial background and resident region of study population, diagnostic criteria, matching, source of samples, genotype detecting method of each study, number of cases and controls, distribution of genotypes and alleles in both case and control groups.

### Statistical analysis

Odds ratios (OR) corresponding to 95% confidence interval (CI) was applied to measure the strength of the association of A-6G and A-20C with hypertension as case–control studies were used, and OR was calculated according to the method of Woolf [25]. We examined the association between allele A of A-6G and hypertension (A vs. G), the dominant genetic model (AA+AG vs. GG), and the recessive genetic model (AA vs. AG+GG). The same method was applied to analyze the A-20C polymorphism. In our study, only the random-effects model using the DerSimonian and Laird's method was employed to bring the individual effect-size estimates together in Review-Manager 5.0.25 software [26]. We then performed a chi-square-based Q statistic test to assess the between-study heterogeneity [27]. Heterogeneity was considered significant for P<0.10 because of the low power of the statistic. The inconsistency index I^2^ was also calculated to evaluate the variation which was caused by heterogeneity rather than by chance, and higher values of the index indicate the existence of heterogeneity [28]. The significance of the pooled OR was determined by the Z test and a P value of <0.05 was considered significant. For each genetic comparison, subgroup analysis according to racial descent was considered for Han Chinese and non-Han Chinese minorities to estimate ethnic-specific OR. In addition, subgroup analysis according to gender was also carried out. Each subgroup had at least two independent studies.

When unexpected heterogeneity was present, sensitivity analysis was performed to examine specific sensitivity of the findings. This analysis was conducted by examining and recalculating the pooled association sizes and joint values of P in homogeneous subgroups, as well as after excluding studies one by one.

Publication bias was investigated by funnel plot, in which the standard error of the log (OR) of each study was plotted against its OR. An asymmetric plot suggested possible publication bias. Funnel-plot asymmetry was assessed by the method of Egger's linear regression test [29]. We performed a t-test to determine the significance of the intercept, and a P-value of <0.05 was considered significant. HWE was tested by the chi-square-test for goodness of fit based on a web program (http://ihg.gsf.de/cgi-bin/hw/hwa1.pl). All statistical analyses were performed using ReviewManager 5.0.25 (Oxford, England) and the software Stata version 10.0 (Stata Corporation, College Station, Texas, USA). All P-values were two-sided.

## Results

### Selection of studies

**Figure 1:**
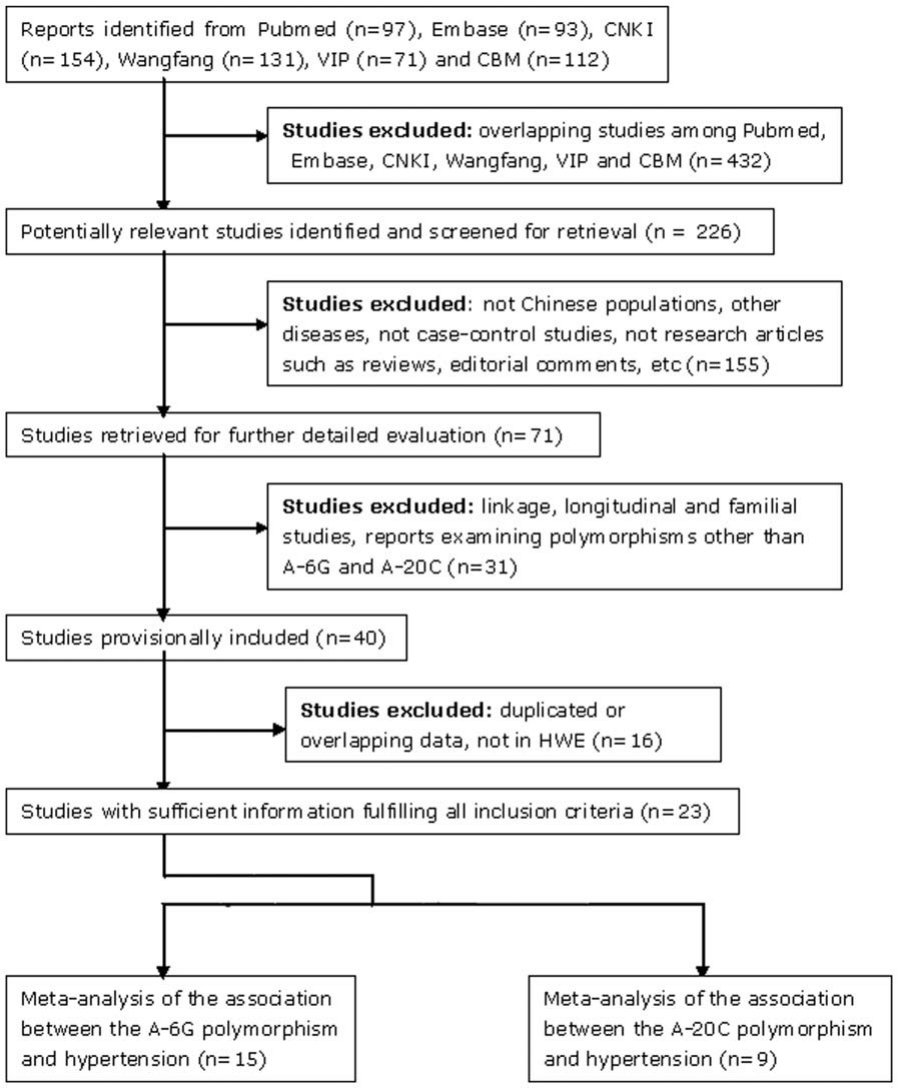
The flowchart of selection of studies and specific reasons for exclusion from the meta-analysis.

After literature search and selection applying our inclusion criteria, we identified a total of 27 relevant articles (40 studies) [7]–[24], [30]–[38]. Among the 40 eligible studies, seven studies [13], [23], [30]–[34] were excluded because they shared the same or overlapping data with others. Moreover, eight studies [13]–[14], [22], [24], [32], [35]–[37] with control groups deviating from HWE were deleted. Finally, 15 studies [7], [9]–[12], [14]–[21], [38] containing 3442 hypertensive patients and 3058 controls for A-6G, as well as 9 studies [7], [11], [15], [17], [20]–[24] containing 2490 hypertensive patients and 2173 controls for A-20C, were collected as being appropriate for the meta-analysis (Figure 1). Thereof, Ge et al. [16], Wang et al. [18] Yue et al. [21] and Liu et al. [38], were four unpublished theses acquired from medical doctorate dissertation database, which was one public sub-database shared by CNKI and Wanfang databases. One paper by Liu et al. [12] provided data on subjects of two Chinese minorities: Tibetan and Yi. Thus, the two minorities were treated as separate studies. For the Yi population, the samples (both cases and controls) were selected only from male individuals. The characteristics of the included studies were summarized in Table 1. Genotype distributions of the control population met HWE for all qualified studies (P>0.05). The flowchart summarizing the process of study selection and reasons for exclusion was presented in Figure 1.

**Table 1:** Detailed characteristics of eligible studies considered in the meta-analysis.

First author	Year	Ethnicity	Region	Single-nucleotide polymorphism	Diagnostic criteria	Matching	Source	Method
Ge [16]	2000	Tibetan	Tibet	A-6G	SBP≥140, DBP≥90	Yes	P-B	PCR-RFLP
Hu [14]	2007	Mongolian	Inner Mongolian	A-6G	SBP≥140, DBP≥90	Yes	P-B	Sequencing technique
Jiang [17]	2009	Han	Jiangsu	A-6G, A-20C	SBP≥140, DBP≥90	Yes	P-B	TaqMan - PCR
Kong [15]	2002	Han	Henan	A-6G, A-20C	SBP≥160, DBP≥95	Yes	H-B	PCR-RFLP
Liu [38]	2002	Han	Shanghai	A-6G	SBP≥140, DBP≥90	No	H-B	Sequencing technique
Li [22]	2004	Kazakh	Xinjiang	A-20C	SBP≥140, DBP≥90	Yes^1^	P-B	PCR-SSCP
Liu [12]	2001	Tibetan	Tibet	A-6G	SBP>140, DBP>90	Yes	P-B	PCR-RFLP
Liu [12]	2001	Yi	Sichuan	A-6G	SBP>140, DBP>90	Yes	P-B	PCR-RFLP
Qi [7]	2008	Han	Beijing	A-6G, A-20C	SBP≥140, DBP≥90	Yes^1^	P-B	PCR-RFLP
Wang [9]	2002	Amis	Taiwan	A-6G	SBP≥140, DBP≥90	Yes	H-B	Sequencing technique
Wang [18]	2003	Kazakh	Xinjiang	A-6G	SBP≥160, DBP≥95	Yes	P-B	MS-PCR
Wang [11]	2007	Li	Hainan	A-6G, A-20C	SBP≥140, DBP≥90	Yes	H-B	Sequencing technique
Wu [10]	2004	Han	Taiwan	A-6G	SBP≥140, DBP≥90	Yes^1^	H-B	Sequencing technique
Yang [23]	2000	Tibetan	Tibet	A-20C	SBP≥140, DBP≥90	Yes	P-B	PCR-RFLP
Yao [19]	2010	Bai	Yunnan	A-6G	SBP≥140, DBP≥90	No	H-B	PCR-RFLP
Ying [20]	2010	Mongolian	Inner Mongolian	A-6G, A-20C	SBP≥140, DBP≥90	No	P-B	PCR-RFLP
Yue [21]	2008	Han	Hebei	A-6G, A-20C	SBP≥140, DBP≥90	Yes2	P-B	PCR-RFLP
Liu [24]	2004	Han	Shanghai	A-20C	SBP>140, DBP>90	No	H-B	Sequencing technique

Abbreviations: SBP, systolic blood pressure (mmHg); DBP, diastolic blood pressure (mmHg); P-B, population-based study; H-B, hospital-based study; PCR-RFLP, polymerase chain reaction and restriction fragment length polymorphism; PCR-SSCP, polymerase chain reaction and single strand conformation polymorphism; MS-PCR, mutagenically separated polymerase chain reaction; Yes, age- and gender- matched, Yes^1^, gender-matched, Yes^2^, age-matched.

### Association between the AGT A-6G polymorphism and hypertension

**Figure 2:**
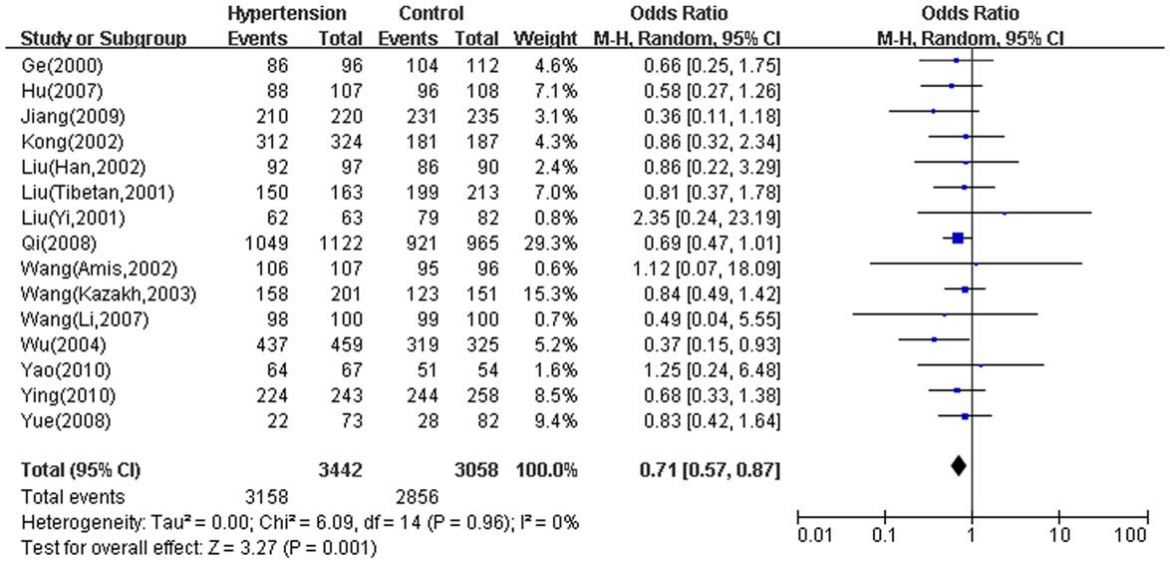
Meta-analysis for the overall association between the A-6G polymorphism and hypertension under the dominant genetic model. Figure 2 shows that the -6A allele carrier (AA+AG) can reduce the risk of hypertension compared to the homozygous GG genotype carriers.

The distribution of AGT A-6G genotypes and alleles in the individual studies was listed in Table 2. The pooled overall frequency of the -6A allele in the Chinese populations was 75.09% in hypertensive cases and 76.60% in normotensive controls. The main results of the meta-analysis about A-6G and the heterogeneity test were presented in Table S1. For all subjects, in the allele comparison and recessive genetic model, there were no significant association between the A-6G polymorphism and hypertension (A vs. G: P = 0.08, OR = 0.9, 95%CI 0.8–1.01, P_heterogeneity_ = 0.09, I^2^ = 35%. AA vs. AG+GG: P = 0.4, OR = 0.93, 95%CI 0.8–1.09, P_heterogeneity_ = 0.04, I^2^ = 43%) (Table S1). However, we detected significantly reduced risk of hypertension in the dominant genetic model (AA+AG vs. GG: P = 0.001, OR = 0.71, 95%CI 0.57–0.87, P_heterogeneity_ = 0.96, I^2^ = 0) (Figure 2).

**Table 2:** Sample size, the distribution of A-6G genotypes and allele frequencies, and P-values of HWE.

	Sample size		AA(genotype)		AG(genotype)		GG(genotype)		A allele frequency (%)		HWE(P* value)
First author	Cases	Controls	Cases	Controls	Cases	Controls	Cases	Controls	Cases	Controls	Controls
Ge [16]	96	112	36	49	50	55	10	8	63.54	68.30	0.1558
Hu [14]	107	108	53	59	35	37	19	12	65.89	71.76	0.1078
Jiang [17]	220	235	136	169	74	62	10	4	78.64	85.11	0.5325
Kong [15]	324	187	200	104	112	77	12	6	79.01	76.20	0.0641
Liu(Han) [38]	97	90	65	59	27	27	5	4	80.93	80.56	0.6878
Liu(Tibetan) [12]	163	213	64	102	86	97	13	14	65.64	70.66	0.2927
Liu(Yi) [12]	63	82	47	52	15	27	1	3	86.51	79.88	0.8259
Qi [7]	1122	965	671	608	378	313	73	44	76.65	79.22	0.6470
Wang(Amis) [9]	107	96	89	65	17	30	1	1	91.12	83.33	0.2207
Wang(Kazakh) [18]	201	151	77	52	81	71	43	28	58.46	57.95	0.6550
Wang(Li) [11]	100	100	83	89	15	10	2	1	90.50	94.00	0.2565
Wu [10]	459	325	316	229	121	90	22	6	82.03	84.31	0.4010
Yao [19]	67	54	33	30	31	21	3	3	73.49	75.00	0.7855
Ying [20]	243	258	138	161	86	83	19	14	74.49	78.49	0.4473
Yue [21]	73	82	3	1	19	27	51	54	80.77	82.32	0.2354

Abbreviations: HWE, Hardy–Weinberg equilibrium.

*The P-value of HWE determined by the χ^2^ test.

In the subgroup analysis by ethnicity, studies were categorized into three groups: Han Chinese, Tibetan and Mongolian. The number of these subpopulations was as follows: six studies involved Han Chinese subjects (2295 cases and 1884 controls), two studies involved Chinese Mongolian (350 cases and 366 controls) and two studies involved Chinese Tibetan (259 cases and 325 controls). The -6A allele had a much higher representation in cases and controls of Han Chinese (76.54% and 77.92%, respectively) than that of Tibetan (64.87 and 69.84%, respectively) and of Mongolian (71.86 and 76.5%, respectively). In Han Chinese population, significant association between the A-6G polymorphism and the decreased risk for hypertension was observed in the dominant genetic model (AA+AG vs. GG: P = 0.005, OR = 0.66, 95%CI 0.50–0.88, P_heterogeneity_ = 0.64, I^2^ = 0)(Table S1). In the allele comparison and recessive genetic model, no evidence of association was found (Table S1). For the subgroups of non-Han Chinese minorities, we found no significant association in any genetic models in the population of Tibetan (Table S1). For Mongolian, a borderline decreased risk of hypertension was seen for A allele carriers compared with G allele carriers (A vs. G: P = 0.05, OR = 0.79, 95%CI 0.62–1.00, P_heterogeneity_ = 0.84, I^2^ = 31%) (Table S1). There was no significant association found in other genetic models conducted using Mongolian (Table S1).

**Figure 3:**
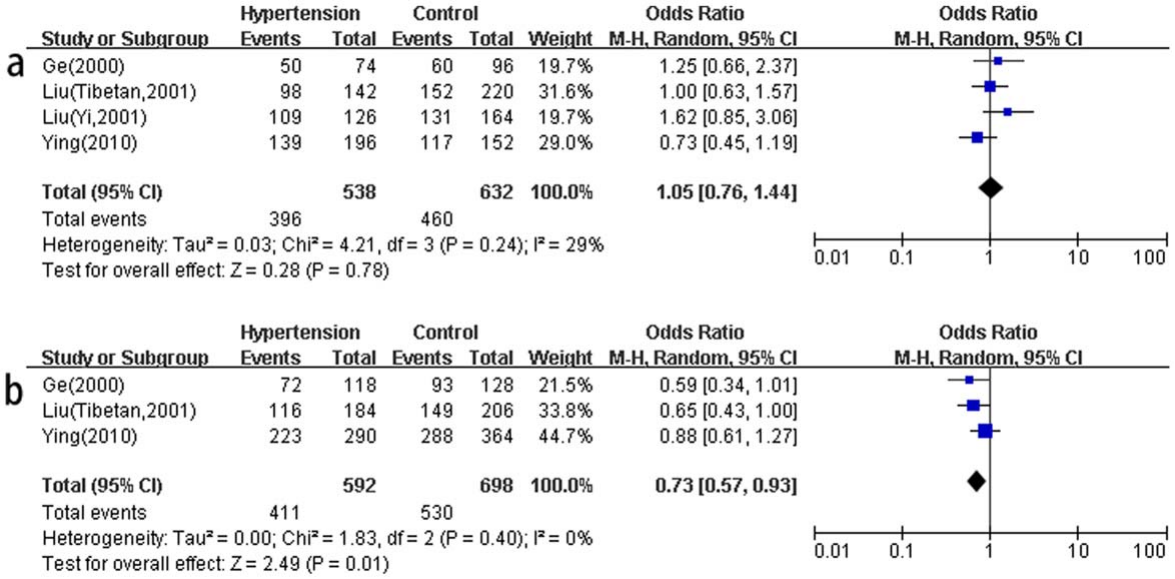
Meta-analysis for the association between the A-6G polymorphism and hypertension under allele comparison (A vs. G) in the subgroup by sex. Figure 3a shows that the A-6G polymorphism is not associated with hypertension in men. Figure 3b shows that the -6 A allele carrier can reduce the risk of hypertension in women compared to the -6G allele carrier.

Considering the fact that the sex discrepancy might bias the overall association, a further subgroup analysis was conducted according to gender. As reflected in Table 3, four studies (3 articles) provided data for males and females, respectively. Accordingly, a total of 269 hypertension patients and 316 controls for males, as well as 296 hypertension patients and 349 controls for females, were investigated. In male subjects, the lack of significant association was found between A-6G and hypertension in all genetic models (A vs. G: P = 0.78, OR = 1.05, 95%CI 0.76–1.44, P_heterogeneity_ = 0.24, I^2^ = 29%) (Figure 3a). However, in female subjects, the A-6G polymorphism displayed significantly reduced risk for hypertension in the allele comparison (A vs. G: P = 0.01, OR = 0.73, 95%CI 0.57–0.93, P_heterogeneity_ = 0.40, I^2^ = 0%) (Figure 3b) and recessive genetic model (AA vs. AG+GG: P = 0.02, OR = 0.69, 95%CI 0.50–0.95, P_heterogeneity_ = 0.49, I^2^ = 0%).

**Table 3:** The characteristics of all included studies for the A-6G polymorphism in the sex-specific subgroup analysis.

				Genotype(Number, M/F)					Blood pressure	
First author	Ethnicity	Status	Number, M/F	AA	AG	GG	Age, year	BMI, kg/m^2^	SBP, mmHg	DBP, mmHg
Ge [16]	Tibetan	Cases	37/59	16/20	18/32	3/7	49.53±11.4	23.34±3.99	159.74±23.15	105.96±10.83
		Controls	48/64	17/32	26/29	5/3	47.98±12.07	21.54±3.11	116.38±16.66	78.21±9.94
Liu(Tibetan) [12]	Tibetan	Cases	71/92	30/34	38/48	3/10	48±12	24±4	164±20	105±12
		Controls	110/103	52/50	48/49	10/4	46±10	22±3	117±14	78±9
Liu(Yi) [12]	Yi	Cases	63/0	47/0	15/0	1/0	51±12	25±4	158±30	103±16
		Controls	82/0	52/0	27/0	3/0	49±6	21±3	112±8	73±6
Ying [20]	Mongolian	Cases	98/145	52/86	35/51	11/8	53.5±11.3	23.2±4.1	159.4±24.2	98.3±11.3
		Controls	76/182	45/116	27/56	4/10	50.3±9.5	22.1±3.2	119.6±10.8	78.1±6.3

Abbreviations: M/F, males/females; BMI, body mass index; SBP, systolic blood pressure; DBP, diastolic blood pressure; Values, mean±s.d.

### Association between the AGT A-20C polymorphism and hypertension

**Figure 4:**
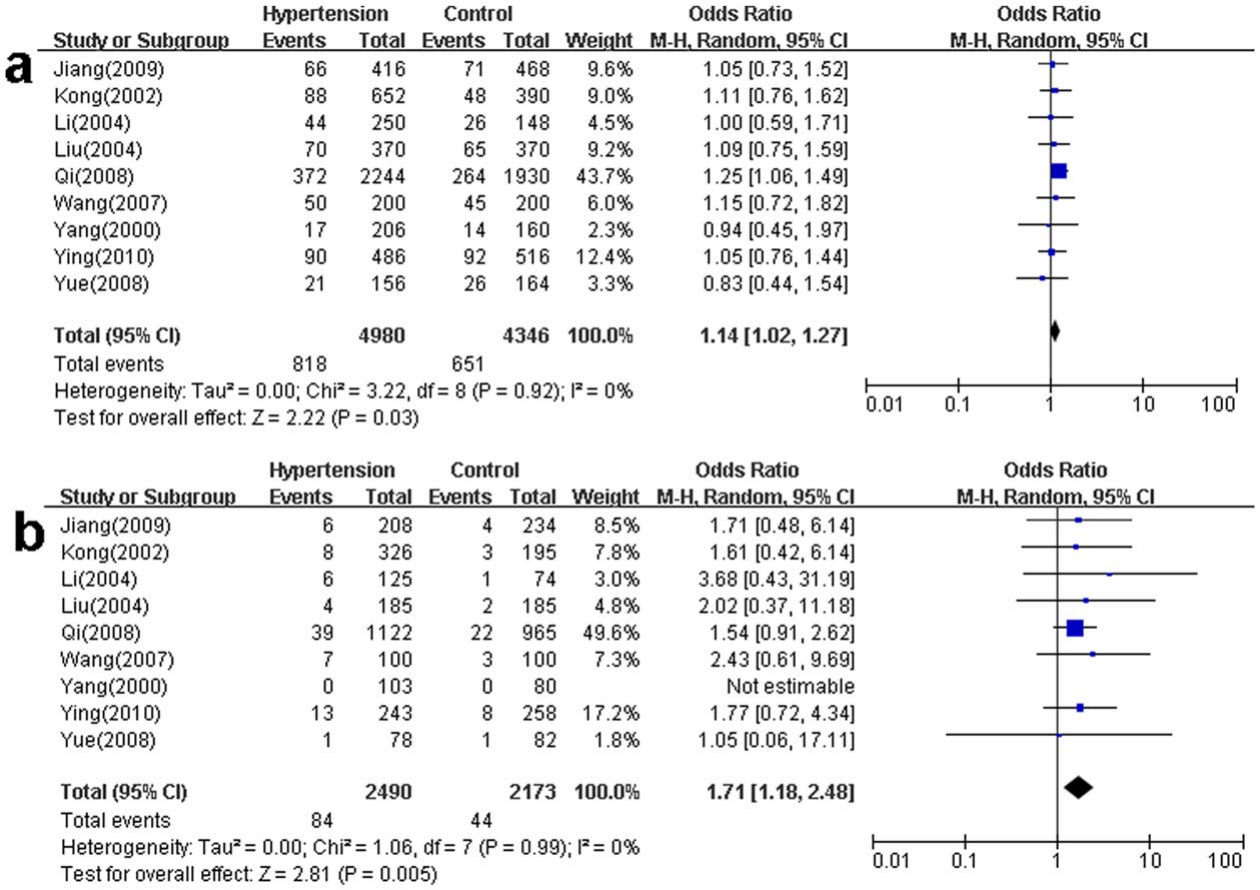
Meta-analysis for the overall association between the A-20C polymorphism and hypertension under various genetic contrasts. Figure 4a shows that the C allele carrier can increase the risk of hypertension compared to the A allele carrier. Figure 4b shows that the homozygous CC genotype carriers can increase the risk of hypertension compared to the A allele carrier (CA+AA).

The distribution of AGT A-20C genotypes and alleles in the individual studies was showed in Table 4. The overall prevalence of the -20C allele in the Chinese populations was 16.43% in cases and 14.98% in controls. The main results of the meta-analysis about A-20C and the heterogeneity test were presented in Table S1. Overall, the significant increased risk of hypertension could be found in the allele comparison (C vs. A: P = 0.03, OR = 1.14, 95%CI 1.02–1.27, P_heterogeneity_ = 0.92, I^2^ = 0) (Figure 4a) and recessive genetic model (CC vs. CA+AA: P = 0.005, OR = 1.71, 95%CI 1.18–2.48, P_heterogeneity_ = 0.99, I^2^ = 0) (Figure 4b). There was no significant association found in the dominant genetic model (CC+CA vs. AA: P = 0.14, OR = 1.10, 95%CI 0.97–1.25, P_heterogeneity_ = 0.83, I^2^ = 0) (Table S1).

**Table 4:** Sample size, the distribution of A-20C genotypes and allele frequencies, and P-values of HWE.

	Sample size		CC(genotype)		CA(genotype)		AA(genotype)		C alleleFrequency(%)		HWE(P* value)
First author	Cases	Controls	Cases	Controls	Cases	Controls	Cases	Controls	Cases	Controls	Controls
Jiang [17]	208	234	6	4	54	63	148	167	15.87	15.17	0.4815
Kong [15]	326	195	8	3	72	42	246	150	13.50	12.31	0.9756
Li [22]	125	74	6	1	32	24	87	49	17.60	17.57	0.3028
Liu [24]	185	185	4	2	62	55	119	128	18.92	15.95	0.1380
Qi [7]	1122	965	39	22	294	220	789	723	16.58	13.68	0.2823
Wang [11]	100	100	7	3	36	39	57	58	25.00	22.50	0.2367
Yang [23]	103	80	0	0	17	14	86	66	8.25	8.75	0.3910
Ying [20]	243	258	13	8	64	76	166	174	18.52	17.83	0.9317
Yue [21]	78	82	1	1	19	24	58	57	13.46	15.85	0.3798

Abbreviations: HWE, Hardy–Weinberg equilibrium.

*The P-value of HWE determined by the χ^2^ test.

Due to the limited studies of non-Han Chinese minorities, the subgroup analysis by ethnicity was only performed for Han Chinese. Specifically, there were five studies dealing with Han Chinese (1919 cases and 1661 controls), and only one study considered Kazakh, Li, Tibetan and Mongolian populations. In the Han population, we found significantly elevated risk of hypertension with the A-20C polymorphism in the allele comparison (C vs. A: P = 0.02, OR = 1.17, 95%CI 1.02–1.33, P_heterogeneity_ = 0.67, I^2^ = 0) and recessive genetic model (CC vs. CA+AA: P = 0.04, OR = 1.58, 95%CI 1.02–2.45, P_heterogeneity_ = 1.00, I^2^ = 0) (Table S1). No positive association was obtained in the dominant genetic model (Table S1). Finally, because only one article [20] provided data for males and females, respectively, we could not perform an additional subgroup analysis based on gender.

### Sensitivity analysis

Significant between-study heterogeneity only existed among all studies in the meta-analysis of the A-6G polymorphism. Sensitivity analyses were conducted by sequentially removing a single study each time to find out the origin of heterogeneity. As a result, the heterogeneity no longer existed for the A-6G polymorphism when four studies were excluded (Liu (Yi) et al. [12]: A vs. G, P_heterogeneity_ = 0.15; Kong et al. [15]: A vs. G, P_heterogeneity_ = 0.16; Jiang et al. [17]: A vs. G, P_heterogeneity_ = 0.16; Wang (Amis) et al. [9]: A vs. G, P_heterogeneity_ = 0.37, AA vs. AG+GG, P_heterogeneity_ = 0.23). In addition, our analysis showed that the corresponding pooled ORs were materially altered with the sequential removal of these four studies (data not shown). The findings revealed that these independent studies might be the main cause of heterogeneity across all subjects.

### Publication bias

**Figure 5:**
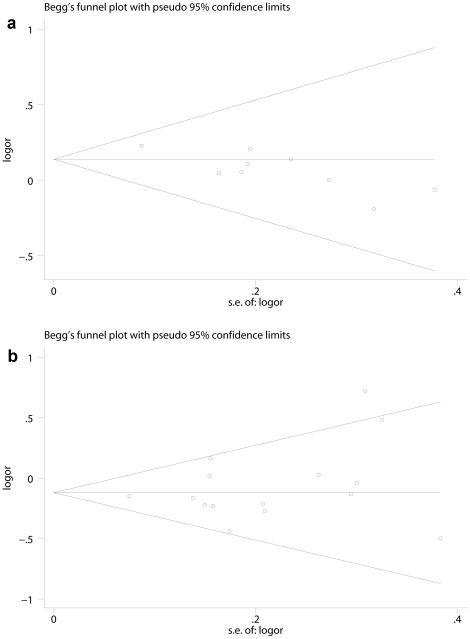
Begg's funnel plot analysis to detect publication bias. Figure 5a shows the funnel plot for allele comparison (A vs. G) of the A-6G polymorphism. Figure 5b shows the funnel plot for allele comparison (C vs. A) of the A-20C polymorphism.

The Egger's test and Begg's funnel plot were applied for allele comparison to asses the publication bias of the literatures. As indicated by the Egger's test and funnel plot, there was no publication bias for the A-6G polymorphism (t = 0.98, P = 0.347 for A vs. G) (Figure 5a), and a possibility of publication bias for the A-20C polymorphism (t = −3.88, P = 0.006 for C vs. A) (Figure 5b).

## Discussion

The literature examining the relationship between the A-6G and A-20C polymorphisms and hypertension in the Chinese populations abounds in small studies reporting controversial findings. No clear consensus has yet been reached. Therefore, we restricted our research to Chinese populations and did a meta-analysis with 24 studies totaling 5932 hypertensive patients and 5231 normotensive controls to form a more precise estimation of their association. To our knowledge, this was one of the largest meta-analysis to date investigating the association of angiotensinogen promoter polymorphisms with hypertension in Chinese. In the current study, we found that the A allele of the A-6G polymorphism was associated with a significant decrease in the risk of hypertension in all subject, and the C allele of A-20C could increase the risk of hypertension. These positive associations should be treated with caution as the P values obtained were reported without correction for multiple testing.

In 1997, Inoue et al. [39] were the first to provide direct evidence for increased transcriptional activity of the -6A variant as compared with the -6G variant in the AGT promoter in a hepatocyte cell line. Since then, much effort has been made to pursue the possible association of the A-6G polymorphism with hypertension. Hegele RA et al. [40] observed that the presence of the AGT -6A variant tended to be associated with higher systolic BP in Canadian Oji-Cree. This positive result was partly confirmed by Ishigami T et al. [41] who studied on Japanese population. A more recent research by Jain S et al. [42] showed that transgenic mice containing -6A haplotype have increased plasma AGT level and increased blood pressure as compared to -6G haplotype, and also reported higher expression of AGT mRNA in Caucasian hypertensive patients carrying the same allele. Moreover, Pereira TV's meta-analysis [8] that came out in 2007 found that A-6G have a higher yet nonsignificant risk for hypertension in Asian populations. The Asian participants included in the analysis were not all from Chinese population, which meant genetic background between this article and our study was different. Nevertheless, these previous findings were inconsistent with the result of our meta-analysis, which was very interesting and exhibited a protective effect on hypertension in the Chinese populations. There were many possible explanations that could be put forward to account for the inconsistency, and ethnic specificity as well as population structure might be the most important potential confounding factors. An obvious supporter was a Chinese case-control study by Liu et al. [12] also generating a protective effect of the A-6G variant on hypertension. It was necessary to perform a meta-analysis in a genetically well-defined population.

Regarding the A-20C polymorphism, a report by Zhao et al. [43] showed that the -20C allele enhanced basal promoter activity on transient transfection in human hepatoma cells (HepG2) as compared with the -20A allele. In human subjects, DR Velez et al. [44] found that this polymorphism seemed to be involved in hypertension in white women. A Chinese case-control article [13] also displayed that the -20C variant could apparently increase the risk of hypertension in the Taiwanese population. Then the results of a 2008 meta-analysis [8] and our meta-analysis verified that the A-20C polymorphism might be major genetic predisposing factor for hypertension in the Chinese populations. Furthermore, with a tight linkage disequilibrium (LD) between the M235T and A-20C polymorphisms, our results about the relationship between A-20C and EH are consistent with previous results by Ji et al. [45] in 2010, which reported that the M235T variant increases the risk of essential hypertension in the Chinese populations (OR = 1.54, 95%CI 1.16–2.03, P = 0.002). An interaction between the A-6G and A–20C variants was biologically plausible [32], [46] because these 2 variants were located in 2 distinct regulatory elements of the core promoter in AGT gene. Subsequently, this interaction between them might affect the transcription of the gene and/or the stability of the resulting mRNA, and in turn play an important role in the pathogenesis of hypertension. Niu et al. [32] observed that the A-6G/A-20C polymorphism was significantly associated with hypertension, which might be attributed to a strong synergistic effect of these two polymorphisms.

China is a huge multi-ethnic country with 56 identified ethnic groups. Among these groups, Han Chinese is the largest ethnic group, making up over 93% of the total population [47]. In the subgroup analysis, we divided studies into two subgroups: Han Chinese and non-Han Chinese minorities. Significant association was identified in Han Chinese for both A-6G and A-20C polymorphisms, which were in accordance with the results in the overall population. For non-Han Chinese minorities, owing to the limited studies and population numbers, only a marginal significant association between A-6G and hypertension was seen in Chinese Mongolian. More studies based on larger population are required to reach more obvious conclusion in different minorities. In another subgroup analysis by gender, the significantly decreased risk of hypertension was associated with the A-6G polymorphism in Chinese women, but not in Chinese men. However, in the view of the relatively small sample size, the conclusion in this sex-specific analysis might be unreliable and must be considered cautiously. Furthermore, no extra subgroup analysis according to gender was undertaken for the A-20C polymorphism because the eligible studies were scarce in number. In the sensitivity analysis, four studies (Wang (Amis) et al. [9], Liu (Yi) et al. [12], Kong et al. [15] and Jiang et al. [17]) were considered as the origin of the heterogeneity. When these articles were deleted, significant association could be found.

The positive findings about A-20C should be interpreted in light of the fact that publication bias was detected. The statistical test and funnel plot inspection in the meta- analysis have indicated the potential for such bias. Publication bias is a relatively common phenomenon in clinical literature [48]–[50], perhaps because positive results have a better chance of being accepted for publication than small studies with non-significant or negative findings. Therefore, conclusions based on these published work might be misleading [51]. In the analysis of the A-20C polymorphism, we conducted a comprehensive search of the published literature and thought it unlikely that many important papers would have been overlooked, but despite this effort, there was still a possibility of publication bias. Thus, the positive findings for A-20C have to be regarded as preliminary.

Several limitations of our meta-analysis need to be noted. First, because of a small number of available studies, we failed to perform additional subgroup analysis in other minority populations (such as Bai), and by gender for the A-20C polymorphism. Second, publication bias was present, and might distort the final conclusion. Third, due to the lack of original data, an evaluation of potential interactions such as gene-gene or gene-environment was not considered in this meta-analysis, which might confound our results.

In conclusion, our meta-analysis suggested significant association between the A-6G and A-20C polymorphisms and hypertension in the Chinese populations, particularly in Han Chinese. More large–scale studies, and especially studies stratified for different minorities and different sexes, should be performed to further elucidate the association between the A-6G and A-20C polymorphisms and hypertension in the Chinese populations.

## Supporting Information

Table S1 **The PRISMA checklist for this meta-analysis.**
(DOC)
