# Supplementary material for: A-6G and A-20C Polymorphisms in the Angiotensinogen Promoter and Hypertension Risk in Chinese: A Meta-Analysis
Source: PLoS One. 2011 Dec 28;6(12):e29489. doi: 10.1371/journal.pone.0029489 (PMC3247271; doi:10.1371/journal.pone.0029489)
Supplement: Table S1 — The PRISMA checklist for this meta-analysis. (DOC) [file pone.0029489.s001.doc]

**Table S1. Summary estimates for ORs and 95% CI in different subgroups under various genetic contrasts.**

| Genotype contrast | Population(study numbers） | P_heterogeneity_ | P* value | OR (95% CI) |
| --- | --- | --- | --- | --- |
| A-6G in hypertension |  |  |  |  |
| A vs.G | Overall(15) | 0.09 | 0.08 | 0.90(0.80-1.01) |
|  | Han(6) | 0.20 | 0.11 | 0.88(0.76-1.03) |
|  | Tibetan(2) | 0.94 | 0.07 | 0.80(0.62-1.02) |
|  | Mongolian(2) | 0.84 | 0.05 | 0.79(0.62-1.00) |
|  | Males(4) | 0.24 | 0.78 | 1.05(0.76-1.44) |
|  | Females(3) | 0.40 | 0.01 | 0.73(0.57-0.93) |
| AA+AG vs. GG | Overall(15) | 0.96 | 0.001 | 0.71(0.57-0.87) |
|  | Han(6) | 0.64 | 0.005 | 0.66(0.50-0.88) |
|  | Tibetan(2) | 0.75 | 0.35 | 0.75(0.41-1.38) |
|  | Mongolian(2) | 0.77 | 0.09 | 0.63(0.37-1.07) |
|  | Males(4) | 0.27 | 0.77 | 1.14(0.49-2.65) |
|  | Females(3) | 0.29 | 0.12 | 0.55(0.26-1.16) |
| AA vs. AG+GG | Overall(15) | 0.04 | 0.40 | 0.93(0.80-1.09) |
|  | Han(6) | 0.13 | 0.46 | 0.93(0.80-1.03) |
|  | Tibetan(2) | 0.79 | 0.06 | 0.73(0.52-1.01) |
|  | Mongolian(2) | 0.93 | 0.14 | 0.80(0.59-1.08) |
|  | Males(4) | 0.30 | 0.85 | 1.04(0.71-1.51) |
|  | Females(3) | 0.49 | 0.02 | 0.69(0.50-0.95) |
| A-20C in hypertension |  |  |  |  |
| C vs. A | Overall(9) | 0.92 | 0.03 | 1.14(1.02-1.27） |
|  | Han(5) | 0.67 | 0.02 | 1.17(1.02-1.33) |
| CC+CA vs. AA | Overall(9) | 0.83 | 0.14 | 1.10(0.97-1.25) |
|  | Han(5) | 0.63 | 0.06 | 1.15(1.00-1.34) |
| CC vs. CA+AA | Overall(9) | 0.99 | 0.005 | 1.71(1.18-2.48) |
|  | Han(5) | 1.00 | 0.04 | 1.58(1.02-2.45) |

Abbreviations: OR, odds ratio; CI, confidence interval; *The P-value of OR determined by the Z test.
